# Immunogenetic Epidemiology of Type 1 Diabetes in 14 Continental Western European Countries

**DOI:** 10.29245/2578-3009/2021/3.1219

**Published:** 2025-08-25

**Authors:** Lisa M. James, Apostolos P. Georgopoulos

**Affiliations:** 1 The HLA Research Group, Brain Sciences Center, Department of Veterans Affairs Health Care System, Minneapolis, MN, 55417, USA; 2 Department of Neuroscience, University of Minnesota Medical School, Minneapolis, MN 55455, USA; 3 Department of Psychiatry, University of Minnesota Medical School, Minneapolis, MN 55455, USA; 4 Department of Neurology, University of Minnesota Medical School, Minneapolis, MN 55455, USA

**Keywords:** Type 1 diabetes, Human leukocyte antigen, Epidemiology, Immunity, Genetics

## Abstract

Human leukocyte antigen (HLA) is widely recognized to influence individual Type 1 diabetes (T1D) risk. Here we utilized an immunogenetic epidemiological approach to evaluate the influence of HLA on T1D at the population level. Specifically, we evaluated the correlations between the population frequencies of 127 HLA Class I and II alleles and the population prevalence of T1D in 14 Continental Western European countries to identify a population-level HLA profile for T1D. The results of these analyses generally corroborated prior findings regarding the influence of HLA on T1D risk and protection and revealed several novel HLA-T1D associations. The findings, discussed within the context of the role of HLA in pathogen elimination and autoimmunity, point to a contributory role of exposure to pathogens in the absence of protective HLA in underlying the autoimmune destruction of pancreatic beta cells in T1D.

## Introduction

Type 1 diabetes (T1D) is a chronic disorder characterized by insulin deficiency and dysregulation of glucose metabolism resulting from destruction of pancreatic beta cells^1^. The majority of T1D cases are autoimmune-mediated^2^. The prevalence of T1D is increasing and varies globally with several European countries (e.g., Finland, Sardinia) exhibiting the highest rates worldwide^3^. The prevailing theory of T1D etiology emphasizes genetic risk coupled with an environmental trigger that results in a pancreatic islet cell autoimmunity and loss of B-cells^4^, although the disease course and progression is variable^1,5^. Several environmental exposures have been associated with T1D. Enteroviruses (e.g., Coxsackievirus B; CVB) are the primary viral candidates for causing type 1 diabetes^1,6^; however, additional viruses (e.g., rotavirus, mumps virus, and cytomegalovirus)^6^ have also been implicated. Other environmental factors including vitamin D, diet, and gut-microbiome diversity have also been shown to influence T1D risk and protection^7^.

In terms of genetic risk, at least half of the genetic risk for T1D is attributed to the human leukocyte antigen (HLA) region on chromosome 6^8^. HLA genes, the most highly polymorphic of the human genome, code for cell-surface proteins that facilitate elimination of foreign antigens. HLA Class I molecules (HLA-A, -B, -C) present intracellular antigen peptides to CD8+ cytotoxic T cells, signaling destruction of infected cells whereas HLA Class II molecules (HLA-DR, DQ, and DP genes) present endocytosed extracellular antigen peptides to CD4+ T cells to promote B-cell mediated antibody production and adaptive immunity. Thus, the evolutionary role of HLA is host protection against foreign antigens; however, HLA is also implicated in a number of autoimmune disorders including T1D^9^. Several HLA Class II alleles, including DRB1 0401, DRB1 0402, DRB1 0405, DQA1 0301, DQB1 0302 or DQB1 0201 alleles and haplotypes, have been associated with T1D risk and protection^10^. Two Class II haplotypes - DRB1*0401-DQB1*0302 and DRB1*0301-DQB1*0201 – have been shown to confer the greatest risk in individuals of European descent whereas others (e.g., DRB1*1501-- DQB1-0602, DRB1*1401- DQB1*0503, and DRB1*0701- DQB1*0303) are protective^11^. Although Class II alleles are most robustly associated with T1D, Class I alleles have also been associated with both T1D risk and protection, even after accounting for linkage disequilibrium effects with Class II alleles^12^. Of the Class I alleles, A*24:02 and HLA-B*39:06 has been most clearly associated with T1D risk and HLA-B*57:01 with protection although numerous other Class I-T1D associations have been identified^12–14^. Notably, more than 60% of T1D patients do not have the highest-risk genotype^15^, highlighting the need to understand risk and protection associated with other HLA alleles.

Here we take an immunogenetic epidemiology approach^16–20^ to identify an HLA profile with regard to T1D disease prevalence to better understand risk and protection associated with a wide range of HLA alleles. Specifically, this approach rests on the correlations between the frequency of HLA alleles in a given population with the population prevalence of a disease. This approach takes advantage of the population heterogeneity of HLA and utilizes high-resolution HLA genotyping to determine HLA alleles that are presumed to be protective (i.e., negatively associated) or susceptible (i.e., positively correlated) with regard to the population prevalence of T1D. In light of the role of HLA in pathogen elimination and autoimmunity, this immunogenetic epidemiology approach provides novel insights into the pathogenesis of T1D at the population-level as well as the variability of T1D prevalence across populations.

## Materials and Methods

### Prevalence of T1D

The population prevalence of T1D was computed for each of the following 14 countries in Continental Western Europe: Austria, Belgium, Denmark, Finland, France, Germany, Greece, Italy, Netherlands, Portugal, Norway, Spain, Sweden, and Switzerland. Specifically, the total number of people with T1D in each of the 14 Continental Western European countries was identified from the Global Health Data Exchange^21,^ a publicly available catalog of data from the Global Burden of Disease study, the most comprehensive worldwide epidemiological study of more than 350 diseases. The Global Health Data Exchange includes epidemiological data separately for Type 1 and Type 2 diabetes; for the present study, only the prevalence data for Type 1 diabetes was obtained. T1D prevalence in each country was divided by the total population of each country in 2016 (Population Reference Bureau)^22^ and expressed as a percentage. We have previously shown that life expectancy for these countries is virtually identical^17^; therefore, life expectancy was not included in the current analyses.

### HLA

**Figure 1.**
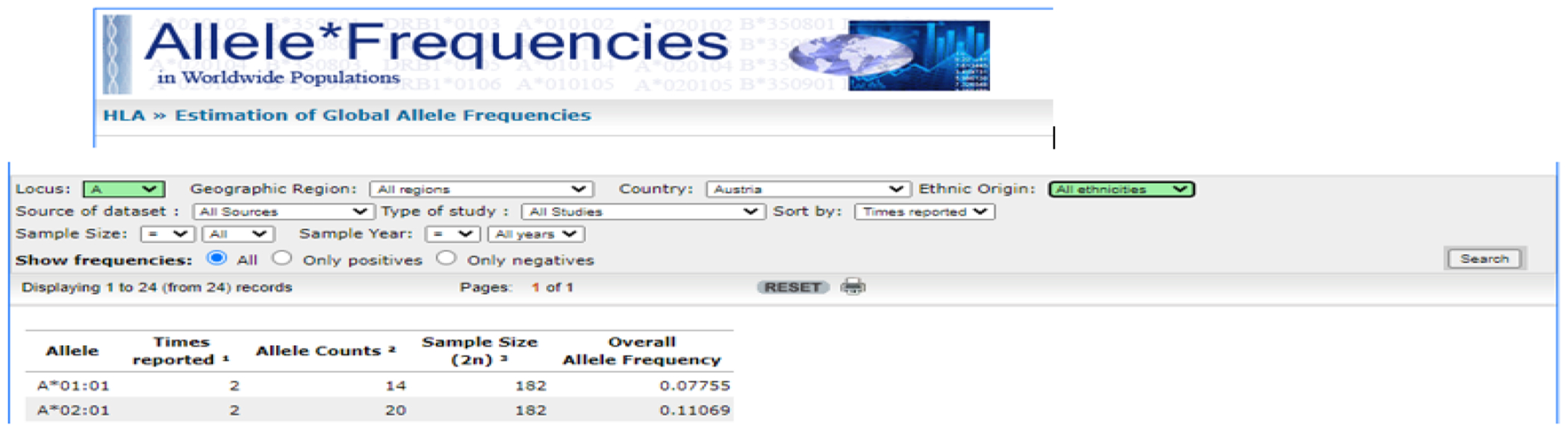
Snapshot of the HLA search performed for each combination of Locus (A, B, C, DPB1, DQB1, DRB1) and Country (14 CWE countries). The Overall Allele Frequency (right-most column) of each Allele (left-most column) was tabulated and analyzed. In the example shown, Locus A and Austria were selected, yielding 24 records, of which the first 2 are shown.

The frequencies of all reported HLA alleles of classical genes of Class I (A, B, C) and Class II (DPB1, DQB1, DRB1) for each of the 14 Continental Western European countries were retrieved from the website allelefrequencies.net (Estimation of Global Allele Frequencies^23,24^) on October 20, 2020. Details of the query are shown in Fig. 1.

There was a total of 2746 entries of alleles from the 14 Continental Western European countries, comprising 844 distinct alleles, i.e., alleles that occurred in at least one country. Of those, 127 alleles occurred in 9 or more countries and were used in further analyses. This criterion is somewhat arbitrary but reasonable; it was partially validated in a previous study^16^, as discussed below.

The distribution of those alleles to the HLA classes and their genes is given in Table 1.

### Data analysis

HLA profiles for T1D were derived as described previously for Parkinson’s disease, dementia, and multiple sclerosis^16,20^. Briefly, the prevalence of T1D in a country was computed as the fraction of total country population and was expressed as a percentage. T1D prevalences were naturallog transformed and the Pearson correlation coefficient, r, between T1D prevalence and the population frequency of each one of the 127 HLA alleles above calculated and Fisher z–transformed^25^ to normalize its distribution:

(1) $${\text{r}}^{\prime }=\text{atanh}(\text{r})$$

The T1D HLA profile consisted of 127 values of ${\text{r}}^{\prime }$. The effects of HLA Class and gene (within a class) on ${\text{r}}^{\prime }$ were evaluated using a univariate analysis of variance (ANOVA). Finally, differences in the proportions of the counts of negative and positive ${\text{r}}^{\prime }$ were evaluated using the Wald H0 statistic for comparing proportions of independent samples. Statistical analyses were performed using the IBM–SPSS package (IBM SPSS Statistics for Windows, Version 26.0, 64–bit edition. Armonk, NY: IBM Corp; 2019) and Intel FORTRAN (Microsoft Visual Studio Community Version 16.8.3; Intel FORTRAN Compiler 2021).

## Results

**Figure 2.**
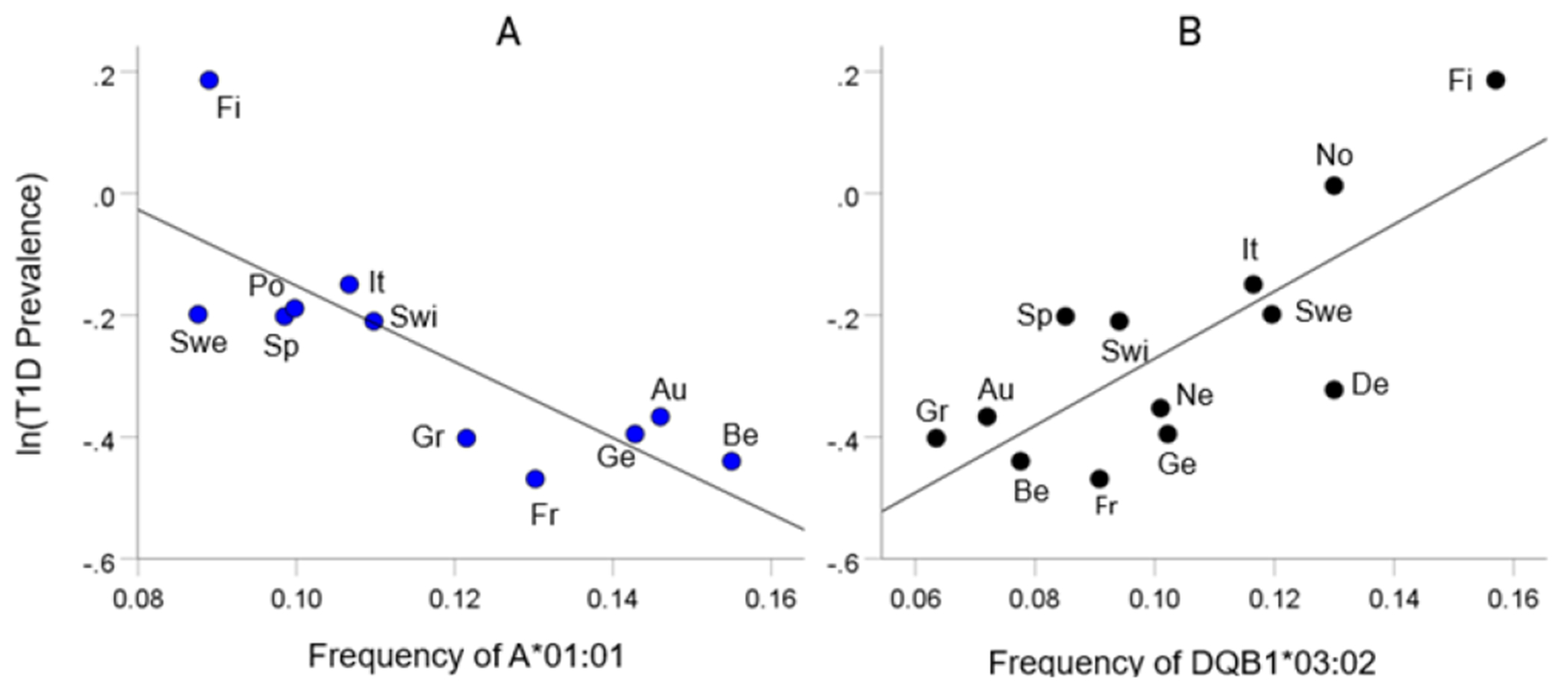
Example from a presumed protective HLA allele (A*01:01) and a presumed susceptibility allele (DQB1*03:02) for T1D. A, log-transformed T1D prevalence (%) for 11 CWE countries is plotted against the corresponding frequency of the A*01:01 (P =0.004). B, log-transformed T1D prevalence (%) for 13 CWE countries is plotted against the corresponding frequency of the DQB1*03:02 (P =0.002). Abbreviations: Au, Austria; Be, Belgium; De, Denmark; Fi, Finland; Fr, France; Ge, Germany; Gr, Greece; It, Italy; Ne, Netherlands; No, Norway; Po, Portugal; Sp, Spain; Swe, Sweden; Swi, Switzerland.

As mentioned above, the T1D HLA profile consists of correlations between allele frequency and disease prevalence, suitably Fisher z-transformed (Equation 1) to normalize their distribution for further analyses. We showed previously^17^ that dementia prevalence varies in an exponential fashion with allele frequency, such that the logarithm of disease prevalence is a linear function of allele frequency. We found the same relation here between T1D prevalence and HLA allele frequency. Two examples are illustrated in Fig. 2, namely for a presumed T1D protective allele (A*01:01) and a susceptibility allele (DQB1*03:02) (Fig. 2A and B, respectively).

### HLA-T1D profile

**Figure 3.**
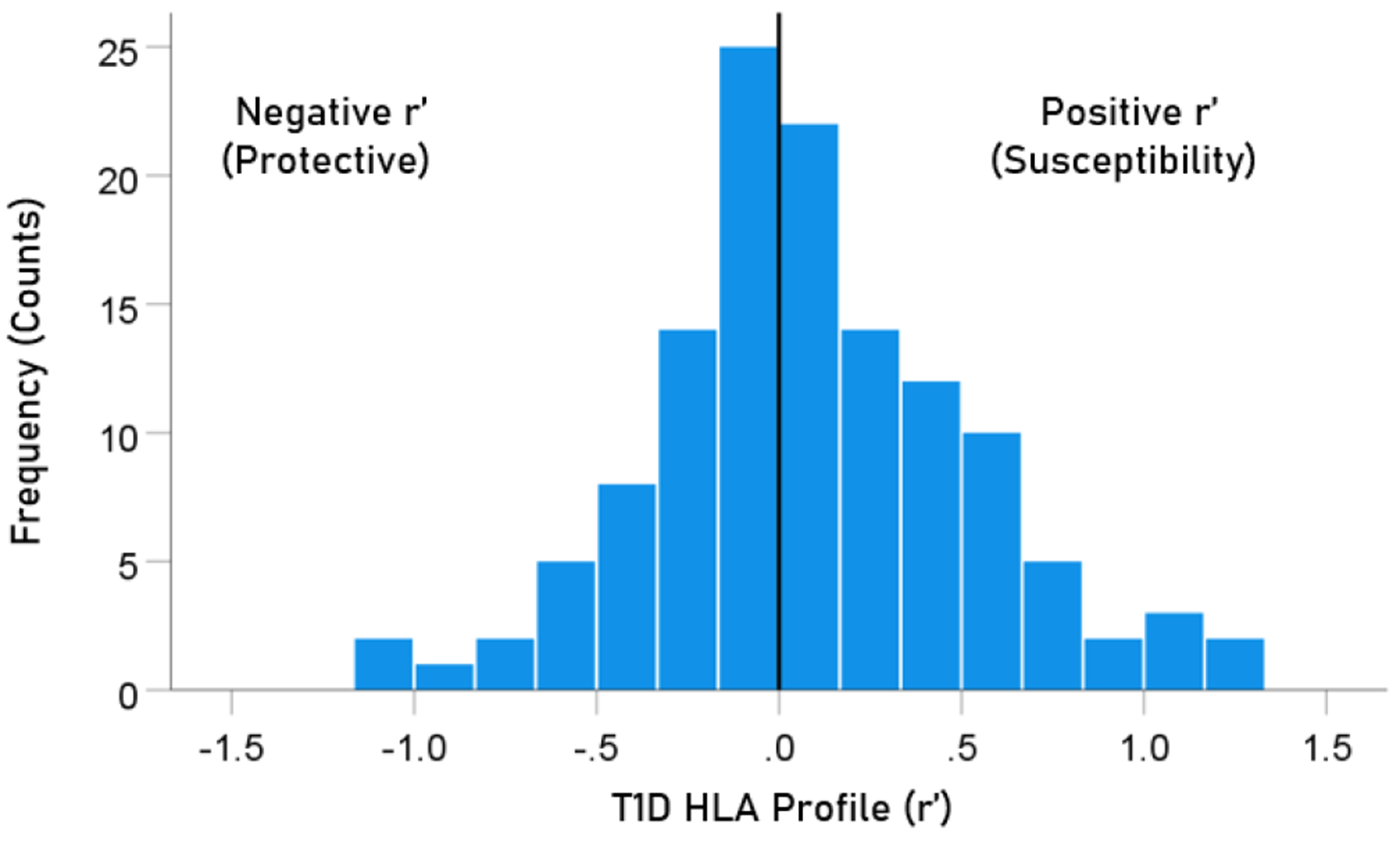
Frequency distribution of T1D HLA profile (N = 127).

The frequency distribution of alleles in the T1D HLA profile (Table 2) is shown in Fig. 3. There were 57/127 (44.9%) negative (protective) alleles and 70/127 (55.1%) positive (susceptibility) alleles. These percentages did not differ significantly from the null hypothesis of 50% (P = 0.249, two-sided one-sample binomial test; z = 1.154).

**Figure 4.**
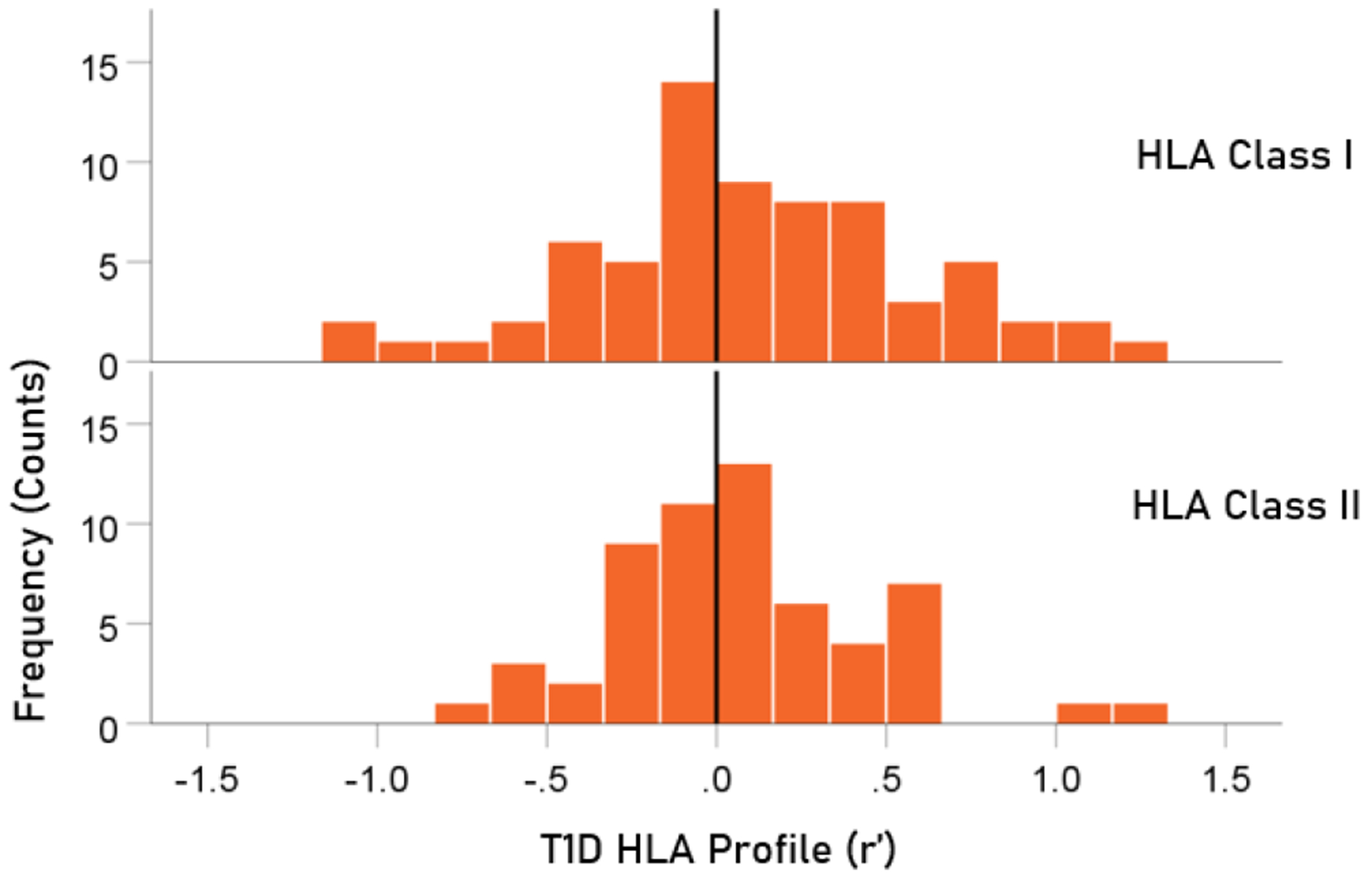
HLA Class distributions of T1D HLA profile. N = 69 alleles for Class I and 58 alleles for Class II.

The distributions of ${\text{r}}^{\prime }$ for Class I and II are shown in Fig. 4. There were 69/127 (54.3%) ${\text{r}}^{\prime }$ in Class I and 58/127 (46.7%) in Class II; these percentages did not differ significantly (P = 0.329, two-sided one-sample binomial test; z = 0.976). For Class I, there were 31/69 (44.9%) negative (protective) and 38/69 (55.1%) positive (susceptibility) values, respectively; these percentages did not differ significantly (P = 0.399, two-sided one-sample binomial test; z = 0.843). For Class II, there were 26/58 (44.8%) negative and 32/58 (55.2%) positive values, respectively; these percentages did not differ significantly (P = 0.431, two-sided one-sample binomial test; z = 0.788).

### Effect of Sample Size

The sample size for estimates of ${\text{r}}^{\prime }$ varied from 9 to 14 (Table 2). We evaluated a possible effect of sample size on ${\text{r}}^{\prime }$ by performing a univariate analysis of variance where ${\text{r}}^{\prime }$ was the dependent variable and the sample size N was random factor. The effect of N was not statistically significant (F_[5,121]_ = 1.185, P = 0.32); in addition, the mean ${\text{r}}^{\prime }$ from any sample did not differ significantly from any other. These results document the lack of bias of sample size on ${\text{r}}^{\prime }$.

### Analysis of Strength of ${\text{r}}^{\prime }$

There were no statistically significant differences in the strength of ${\text{r}}^{\prime }\left(\left|{\text{r}}^{\prime }\right|\right)$ between the negative (protective) and positive (susceptibility) groups for either HLA class or gene (within a class) (P>0.05 for all comparisons, independent samples t-test).

## Discussion

In an extension of our previous work on HLA-disease profiles^16–20^, we used an immunogenetic epidemiological approach across 14 countries in Continental Western Europe to identify a population–level HLA profile consisting of protective and susceptibility alleles for T1D. We used the Fisher z-transformed correlation ${\text{r}}^{\prime }$ between T1D prevalence and HLA allele frequency as a continuous, signed measure of association, where the sign of ${\text{r}}^{\prime }$ indicates the direction of association (negative/protective, positive/susceptibility) and its absolute value |r′| indicates the strength of association. We computed ${\text{r}}^{\prime }$ for samples sizes of N ≥ 9 countries, a reasonable threshold. Although estimates of ${\text{r}}^{\prime }$ from smaller sample sizes gave robust outcomes for the association of HLA profiles of dementia and Parkinson’s disease (see Table 7 in ref.^16^), we deemed it appropriate to stay with the more conservative threshold of N ≥ 9 countries; for the sample sizes used of 9-14 countries, there were no statistically significant differences between estimates of ${\text{r}}^{\prime }$.

The results are generally consistent with extant literature regarding HLA associations with T1D and identify several novel HLA-T1D associations. Of the 127 HLA alleles, 70 were positively associated with the population prevalence of T1D and are presumed to reflect population-level susceptibility. The strongest positive associations with T1D prevalence were found for HLA-A*02:05, DRB1*12:01, A*36:01, A*33:01, and DQB1*03:02, in that order, as shown in Table 2. DQB1*03:02 is part of a haplotype that has well-established associations with T1D^11^. Other alleles associated with high-risk T1D haplotypes (e.g., DRB1*0401, DRB1*0301, DQB1*0201) were also positively associated with the population prevalence of T1D here albeit less so. Previous research has shown that DPB1*03:02 and DPB1*02:02 also increased risk of T1D^15^. Consistent with those findings, both of those alleles were positively associated with T1D prevalence in the current study. Similarly, of the Class I alleles, HLA-B*39:06 has been shown to be strongly associated with T1D risk^12^. Here, too, the association of B*39:06 and the population prevalence of T1D was positive, though several other Class I alleles were more strongly associated with risk for T1D.

Several HLA alleles were also found to be negatively associated with the population prevalence of T1D and are presumed to be protective. The most protective alleles in this population-level study were A*01:01, C*07:04, C*06:02, DPB1*13:01, and A*26:01, in that order, as shown in Table 2. Previous studies have similarly found protective effects for three of these five protective alleles - A*01:01^14^ and C*06:02 and C*07:04^12^. A large international collaborative study demonstrated protective effects of numerous Class I alleles with the strongest protective effects observed for A*11:01, B*07:02, B*44:03, B*57:01, C*04:01, C*07:02, and C*16:01^12^. With two exceptions (C*04:01 and C*07:02), we also found these alleles to be protective. Finally, prior studies have identified protective Class II haplotypes including DRB1*1501--DQB1-0602; DRB1*1401- DQB1*0503, and DRB1*0701- DQB1*0303^11^. In this population-level study we did not evaluate the influence of haplotypes on T1D; nonetheless, our population immunogenetic approach similarly found protective effects of DRB1*14:01, DQB1*0503, and DRB1*07:01 alleles on T1D prevalence.

The protective and susceptibility effects identified here point to population-level immunogenetic influences on T1D and add to the literature in terms of identifying several Class I and Class II HLA alleles that are associated with population-level immunogenetic susceptibility to T1D. However, T1D is thought to result (in most cases) from genetic susceptibility coupled with an uncertain environmental trigger. Given the evolutionary role of HLA in host protection from foreign antigens such as viruses and bacteria, the present findings documenting several strong positive and negative HLA associations with T1D implicate exposure to pathogens as a potential trigger in T1D. Indeed, a number of viruses have been implicated in T1D including, most prominently, enteroviruses^6^. We presume that protective alleles identified here facilitate pathogen elimination that could otherwise contribute to T1D whereas susceptibility alleles promote autoimmune destruction of pancreatic beta cells through an uncertain mechanism that could include viral persistence or molecular mimicry with viral epitopes^26^. Certainly, non-viral population-level environmental factors such as diet may also contribute to T1D. Population level differences in pathogen exposure, diet, or other environmental factors coupled with geographic and ethnic variability in HLA^27,28^ may partially account for the global difference in T1D prevalence.

There are several strengths of the immunogenetic epidemiological approach used here. The ability to evaluate the association of a large number of high–resolution Class I and Class II HLA alleles with T1D prevalence across countries is a significant strength of the current study as low–resolution HLA genotyping has been shown to mask important protein–level differences in HLA-T1D associations^15^ and reliance on a single country may limit allelic variation and reduce regional generalizability. An additional strength of this approach is that population-level analyses permit large-scale HLA-disease association analyses that minimize the influence of individual difference variables that may otherwise confound HLA-disease association studies in individuals or cohorts. Finally, this immunogenetic epidemiological approach is beneficial for identifying immunogenetic contributions to population-level disease variability. The applicability of these population-level findings to individual T1D risk and protection await validation; however, given the heterogeneity of HLA, prohibitively large samples are typically needed for cohort studies aimed at evaluating the presence or absence of a large number of HLA genes in individuals with T1D compared to healthy controls.

Despite strengths of this immunogenetic epidemiological study of T1D, several qualifications and limitations must also be considered. First, the HLA–T1D associations observed in these 14 Continental Western European countries may not extend to other countries within Western Europe that were not investigated here or to other regions. Indeed, HLA-T1D associations have been shown to vary geographically^3,15^; thus, a similar population-level approach in different regions will be useful for determining regional similarities or differences in HLA-T1D associations. Such analyses are underway in our lab. Second, several haplotypes have been associated with individual risk for T1D^11,15^. Since our study was aimed at population-level analyses, we focused instead on the influence of the population frequency of individual alleles on T1D prevalence. Overall, the direction of effects in terms of susceptibility or protection were remarkably similar in our population-level findings and previous disease-association studies evaluating allele or haplotype associations with T1D. Third, the current study is focused on T1D prevalence; the extent to which HLA is associated with other epidemiological T1D measures such as incidence and mortality remain to be investigated. Furthermore, the prevalence data used here assumes accurate classification of T1D; however, it is possible that T1D prevalence is misestimated due to misclassification of T1D as Type 2 diabetes^29^. Finally, T1D is generally thought to result from an environmental trigger coupled with genetic susceptibility. Although HLA associations with T1D implicate pathogens as relevant environmental triggers, evaluation of specific pathogenic influences is beyond the scope of the current paper. Similarly, since the present study was specifically aimed at evaluating the contribution of HLA to T1D, the influence of other genetic^30^ and environmental factors was not evaluated in the present study. Certainly, HLA is one of many factors contributing to variability in T1D prevalence.

## Conclusion

It is well established that HLA significantly influences individual risk for T1D. Here we evaluated immunogenetic influences on T1D at the population level. The findings were highly similar with the extant literature regarding individual susceptibility and protective alleles and several novel HLA-T1D associations were identified. In light of the role of HLA in pathogen elimination and autoimmunity, these findings point to a contributory role of exposure to pathogens in the absence of protective HLA as partially underlying T1D.

## Figures and Tables

**Table 1.** Distribution of 127 HLA alleles analyzed to Class and Genes.

	Class I (N = 69 alleles)			Class II (N = 58 alleles)		
Gene	A	B	C	DPB1	DQB1	DRB1
Count	20	36	13	15	14	29

**Table 2.** HLA profile of T1D. The signed z-transformed correlation coefficient $\left({\text{r}}^{\prime }\right)$ between 127 HLA alleles and ln(T1D) prevalence. N denotes the number of CWE countries from which ${\text{r}}^{\prime }$ was calculated.

	Allele	Class	N	${\text{r}}^{\prime }(\text{T1D})$
1	A*01:01	I	11	−1.064
2	A*02:01	I	11	0.280
3	A*02:05	I	9	1.317
4	A*03:01	I	11	0.477
5	A*11:01	I	11	−0.012
6	A*23:01	I	11	−0.156
7	A*24:02	I	11	0.205
8	A*25:01	I	12	−0.088
9	A*26:01	I	11	−0.677
10	A*29:01	I	11	0.550
11	A*29:02	I	11	0.088
12	A*30:01	I	11	−0.163
13	A*30:02	I	12	0.036
14	A*31:01	I	9	0.203
15	A*32:01	I	12	−0.386
16	A*33:01	I	10	1.032
17	A*33:03	I	9	−0.068
18	A*36:01	I	10	1.058
19	A*68:01	I	11	−0.295
20	A*68:02	I	10	−0.091
21	B*07:02	I	10	−0.153
22	B*08:01	I	12	−0.361
23	B*13:02	I	11	−0.392
24	B*14:01	I	11	0.697
25	B*14:02	I	10	0.861
26	B*15:01	I	10	0.092
27	B*15:17	I	9	0.499
28	B*15:18	I	9	−0.065
29	B*18:01	I	12	0.009
30	B*27:02	I	10	0.655
31	B*27:05	I	12	0.177
32	B*35:01	I	11	0.723
33	B*35:02	I	9	0.324
34	B*35:03	I	9	0.054
35	B*35:08	I	9	0.020
36	B*37:01	I	10	0.150
37	B*38:01	I	9	0.604
38	B*39:01	I	11	−0.061
39	B*39:06	I	9	0.360
40	B*40:01	I	12	0.375
41	B*40:02	I	12	0.054
42	B*41:01	I	11	0.444
43	B*41:02	I	10	0.254
44	B*44:02	I	12	−0.537
45	B*44:03	I	12	−0.176
46	B*44:05	I	9	0.067
47	B*45:01	I	10	−0.114
48	B*47:01	I	11	0.481
49	B*49:01	I	11	0.428
50	B*50:01	I	10	0.827
51	B*51:01	I	10	−0.136
52	B*52:01	I	10	−0.214
53	B*55:01	I	11	−0.211
54	B*56:01	I	9	0.828
55	B*57:01	I	12	−0.380
56	B*58:01	I	9	0.394
57	C*01:02	I	9	0.939
58	C*03:03	I	9	0.729
59	C*04:01	I	9	0.280
60	C*05:01	I	9	−0.093
61	C*06:02	I	9	−0.916
62	C*07:01	I	9	−0.630
63	C*07:02	I	9	0.326
64	C*07:04	I	9	−1.020
65	C*12:02	I	9	−0.337
66	C*12:03	I	9	−0.315
67	C*14:02	I	9	−0.443
68	C*15:02	I	9	−0.004
69	C*16:01	I	9	−0.049
70	DPB1*01:01	II	11	0.596
71	DPB1*02:01	II	11	−0.198
72	DPB1*02:02	II	10	0.132
73	DPB1*03:01	II	11	0.331
74	DPB1*04:01	II	11	0.066
75	DPB1*04:02	II	11	−0.075
76	DPB1*05:01	II	11	0.557
77	DPB1*06:01	II	10	−0.047
78	DPB1*09:01	II	9	0.427
79	DPB1*10:01	II	10	−0.352
80	DPB1*11:01	II	9	0.337
81	DPB1*13:01	II	10	−0.790
82	DPB1*14:01	II	11	−0.282
83	DPB1*17:01	II	9	−0.038
84	DPB1*19:01	II	11	0.158
85	DQB1*02:01	II	12	0.176
86	DQB1*02:02	II	11	−0.132
87	DQB1*03:01	II	13	−0.648
88	DQB1*03:02	II	13	1.031
89	DQB1*03:03	II	13	0.233
90	DQB1*04:02	II	13	0.501
91	DQB1*05:01	II	13	−0.297
92	DQB1*05:02	II	10	−0.123
93	DQB1*05:03	II	12	−0.111
94	DQB1*06:01	II	11	−0.503
95	DQB1*06:02	II	14	0.198
96	DQB1*06:03	II	13	0.129
97	DQB1*06:04	II	12	−0.559
98	DQB1*06:09	II	9	−0.084
99	DRB1*01:01	II	14	0.092
100	DRB1*01:02	II	11	0.578
101	DRB1*01:03	II	11	0.056
102	DRB1*03:01	II	13	0.031
103	DRB1*04:01	II	13	0.131
104	DRB1*04:02	II	11	0.183
105	DRB1*04:03	II	12	0.021
106	DRB1*04:04	II	13	0.160
107	DRB1*04:05	II	9	0.563
108	DRB1*04:07	II	12	0.058
109	DRB1*04:08	II	9	0.547
110	DRB1*07:01	II	12	−0.369
111	DRB1*08:01	II	13	0.553
112	DRB1*08:03	II	11	0.089
113	DRB1*09:01	II	12	0.225
114	DRB1*10:01	II	14	−0.083
115	DRB1*11:01	II	14	−0.281
116	DRB1*11:02	II	12	0.443
117	DRB1*11:03	II	12	−0.231
118	DRB1*11:04	II	12	−0.120
119	DRB1*12:01	II	13	1.177
120	DRB1*13:01	II	14	0.381
121	DRB1*13:02	II	14	−0.266
122	DRB1*13:03	II	10	−0.194
123	DRB1*13:05	II	10	−0.142
124	DRB1*14:01	II	14	−0.126
125	DRB1*15:01	II	13	0.059
126	DRB1*15:02	II	10	−0.214
127	DRB1*16:01	II	10	−0.260
